# Human Induced Pluripotent Stem Cell-Derived Non-Cardiomyocytes Modulate Cardiac Electrophysiological Maturation Through Connexin 43-Mediated Cell-Cell Interactions

**DOI:** 10.1089/scd.2019.0098

**Published:** 2020-01-10

**Authors:** Sherri M. Biendarra-Tiegs, Daniel J. Clemens, Frank J. Secreto, Timothy J. Nelson

**Affiliations:** ^1^ Department of Molecular Pharmacology and Experimental Therapeutics, Mayo Clinic, Rochester, Minnesota.; ^2^ Center for Regenerative Medicine, Mayo Clinic, Rochester, Minnesota.; ^3^ Division of General Internal Medicine, Department of Internal Medicine, Mayo Clinic, Rochester, Minnesota.; ^4^ Department of Cardiovascular Medicine, Mayo Clinic, Rochester, Minnesota.; ^5^ Division of Pediatric Cardiology, Department of Pediatric and Adolescent Medicine, Mayo Clinic, Rochester, Minnesota.

**Keywords:** induced pluripotent stem cells, cardiac, differentiation, cell interactions

## Abstract

The functional maturation status of human induced pluripotent stem cell-derived cardiomyocytes (hiPSC-CMs) has a notable impact upon their use in pharmacological studies, disease modeling, and therapeutic applications. Non-cardiomyocytes (non-CMs) produced in the differentiation process have previously been identified as having an extrinsic influence upon hiPSC-CM development, yet the underlying mechanisms are not fully understood. Herein, we aimed to modulate electrophysiological properties of hiPSC-CMs within co-cultures containing varied proportions of non-CMs and investigate the nature of interactions between these different cell types. Therefore, we sorted cardiac differentiations on day 10 and subsequently replated the cells at ratios of 7:3, 1:1, 3:7, and 1:9 non-CMs to CMs. After a month of co-culture, we evaluated electrophysiological properties through the genetically encoded voltage indicator ArcLight. We ultimately identified that co-cultures with approximately 70%–90% CM purity demonstrated the highest action potential (AP) amplitude and maximum upstroke velocity by day 40 of differentiation, indicative of enhanced electrophysiological maturation, as well as more ventricular-like AP morphologies. Notably, these findings were distinct from those observed for co-cultures of hiPSC-CMs and dermal fibroblasts. We determined that the co-culture phenotypes could not be attributed to paracrine effects of non-CMs due to the inability of conditioned media to recapitulate the observed effects. This led to the further observation of a distinctive expression pattern of connexin 43 (Cx43) at cell-cell interfaces between both CMs and non-CMs. Depletion of Cx43 by short hairpin RNA (shRNA) specifically in the non-CM population within a co-culture environment was able to recapitulate electrophysiological phenotypes of a purer hiPSC-CM population. Collectively, our data demonstrate that abundant non-CM content exerts a significant negative influence upon the electrophysiological maturation of hiPSC-CMs through Cx43-mediated cell-cell-contacts, and thus should be considered regarding the future production of purpose-built hiPSC-CM systems.

## Introduction

Human induced pluripotent stem cells have become widely recognized as a renewable and valuable source of cardiomyocytes (CMs) in vitro, providing a model system for drug screening, disease modeling, and regenerative medicine [1,2]. However, these applications can be hampered by notable variability and heterogeneity in the properties of the human induced pluripotent stem cell-derived cardiomyocytes (hiPSC-CMs) [3]. Some of this can be attributed to the maturation status of the cells, which influences gene expression, electrophysiology, calcium handling, contractility, cell morphology, metabolism, and proliferation [4,5].

Electrophysiological maturation is accompanied by changes in action potential (AP) properties such as increased AP amplitude and maximum upstroke velocity (V_max_) [6]. Furthermore, standard cardiac differentiations have frequently been reported to produce cells with heterogenous AP profiles, which can resemble the characteristic phenotypes of ventricular, atrial, and nodal CM subtypes [7,8]. Being able to understand and appropriately modulate these properties is highly relevant for many uses of hiPSC-CMs. For example, cell therapy with hiPSC-CMs, which more closely resemble the electrophysiological properties of the recipient chamber, may reduce the risk of arrhythmia [9]. Similar considerations can also apply to drug screening and disease modeling purposes.

Adding to this complexity, cardiac differentiations from hiPSCs typically result in a mixed population of CMs and non-CMs. Since these non-CMs have been considered a hindrance to obtaining a robust yield of CMs, varied approaches have been developed to purify hiPSC-CMs [10]. However, there is now evidence that these non-CMs actually influence human pluripotent stem cell-derived CM properties in a variety of ways. For example, one group of researchers found that BRAF-mutant fibroblast-like non-CMs promoted CM hypertrophy phenotypes in hiPSC-CMs by means of paracrine TGFβ signaling [11].

In addition, Kim et al. demonstrated that nearly complete elimination of non-CMs from the cardiac differentiation of embryonic stem cells resulted in arrested maturation of electrophysiology and calcium handling in the resultant CMs. Reintroduction of the non-CMs reversed this stunted functional development [12].

Interestingly, an examination of the properties associated with engineered cardiac tissues containing 25%, 50%, 70%, or 90% hiPSC-CMs revealed that achieving high CM purity (90%) results in functional advantages, including high conduction velocity, increased rising and falling slopes for calcium transients, and a greater percentage of MLC2v^+^/MLC2a^−^ CMs. The study also observed the highest rising and falling AP slopes, maximal capture pacing rate, contraction/relaxation velocities, and contraction/relaxation deformation distances for the 70% CM constructs. Ultimately, the authors concluded that engineered cardiac tissues with 50%–70% CMs held the most potential for regenerative therapies [13].

Altogether, these findings demonstrate that non-CMs can influence multiple aspects of hiPSC-CM maturation and functionality. In addition, the variation in MLC2v and MLC2a expression between different co-culture conditions suggests that non-CMs have the potential to modulate at least one property associated with ventricular maturation.

Given the relevance of hiPSC-CM maturation status and cardiac subtype-directed development for the utilization of these cells, it is necessary to gain a more complete understanding of the impact that non-CMs have upon hiPSC-CMs. Furthermore, previous electrophysiological studies have not focused deeply on exploring the mechanisms underlying the interactions between these different cell types. This knowledge would aid in tailoring hiPSC-CM purity, and thereby functional properties, for a given application. In addition, it would help to further discern the relationship between in vitro environmental factors and functional properties of differentiated progeny from hiPSCs.

The goal of this study was to evaluate the effect of non-CMs on hiPSC-CM electrophysiological maturation and AP shape with extended co-culture. Furthermore, our aim was to determine the means by which non-CMs interact with hiPSC-CMs and thus influence hiPSC-CM electrophysiology. By applying the genetically encoded voltage indicator ArcLight to examine the effect of varied proportions of non-CMs to hiPSC-CMs in co-cultures, we were able to demonstrate the ability of abundant non-CMs to hinder electrophysiological maturation and the development of ventricular-like AP morphologies compared to cultures with greater than ∼50% CM content. Furthermore, we were able to determine that these effects are dependent upon connexin 43 (Cx43)-mediated cell-cell interactions.

Overall, these results indicate that non-CMs are an important consideration in understanding and modulating hiPSC-CM functional properties for translational applications.

## Materials and Methods

A detailed description of additional experimental procedures can be found in the Supplementary Data.

### Cardiac differentiation of hiPSCs in monolayers

hiPSCs were dissociated to a single-cell suspension using TrypLE Express (ThermoFisher) and cultured twice in monolayer stages for 2 days each before initiation of cardiac differentiation, according to our standard protocol. Specifically, hiPSCs were first plated as a monolayer of 6 million cells per 60 mm plate to further expand cell number, and then plated at 60,000 cells per well of a 96-well plate for differentiation. Twenty micromolars ROCK inhibitor was used for plating.

hiPSCs were differentiated to CMs in RPMI-1640 with B-27 supplement minus insulin. Cardiac induction was performed in confluent Geltrex^®^ (A1413301; ThermoFisher)-coated 96-well plates with CHIR99021 (S1263; Selleckchem) and 10 ng/mL of recombinant activin A (338-AC-01M/CF; R&D Systems) for 20 h. CHIR99021 concentrations used were 6, 8, 10, or 12 μmol/L, depending on what successfully produced hiPSC-CMs for a particular clone. On day 3 postinduction, the media were changed to fresh media with 5 μmol/L IWP2 (TOCRS 3533) and 10 ng/mL recombinant human BMP-4 (314-BP-010; R&D Systems) for 2 days. Media with B-27 supplement (containing insulin) were used starting on day 7. Media were replaced every 2–3 days.

### Cardiac differentiation of hiPSCs in spinner culture

Thawed spinner culture hiPSC-CMs from cryopreserved stocks were used for Fig. 6C–E. Spinner culture hiPSC-CMs were produced and cryopreserved by ReGen Theranostics (Rochester, MN). For transition to spinner culture, hiPSCs were passaged using Accutase (EMD Millipore) and 10 μM ROCK inhibitor (Y-27632 dihydrochloride, Tocris 1254) from 60 mm plates to a spinner flask at 300,000 cells/mL using an equal mixture of Stempro (A1000701; ThermoFisher) and mTeSR1. The CELLSPIN system platform and 250 mL flasks were used (183001, 182026; Argos). The stir plate was set to 60 rpm. Fifty percent of media was changed the next day, and 80% on subsequent days. Cells were split using Accutase on the fourth day or if cell aggregates exceeded 300 μm in diameter.

Differentiation was initiated 2 days postsplit by the addition of RPMI-1640 media supplemented with B-27 minus insulin and 8 μmol/L CHIR99021. After 24 h, the media were changed to fresh RPMI-1640 supplemented with B-27 minus insulin. On day 3 postinduction, the cells were treated with fresh media with 5 μmol/L IWP4 (04-0036; Stemgent) for 2 days. On day 5, this was replaced with media without IWP4. On day 7, the media were switched to RPMI-1640 with B-27 supplement (containing insulin). The media were subsequently changed every other day.

CMs from spinner culture differentiations were frozen on day 20 postinduction. Cell aggregates were dissociated to single cells using Accutase, and the cells were subsequently cryopreserved in CryoStorCS10. Spinner CMs were thawed in Cardiomyocyte Maintenance Medium (Gibco) plus 20% fetal bovine serum (FBS) and plated in Cardiomyocyte Maintenance Medium on fibronectin-coated (F1141; Sigma) Nunc optical-bottom 96-well black-walled plates. The cells were maintained in Cardiomyocyte Maintenance Medium until co-culture formation.

### Isolation of single cells derived from hiPSCs, cell sorting, and co-culture formation

hiPSC differentiations on day 10 were dissociated to single cells using 0.05 U of TH Research Grade Liberase (5401135001; Sigma) and 6.3 U of DNase (D4513-1VL; Sigma) per well of a 96-well plate, followed by treatment with TrypLE Express. A similar process was used to collect spinner culture-derived CMs on day 24 (4 days post-thaw). Cells were sorted using the PSC-Derived Cardiomyocyte Isolation Kit, human (130-110-188; Miltenyi Biotec) and the MidiMACS system (Miltenyi Biotec), according to the manufacturer's instructions. Only magnetic labeling of PSC-derived non-CMs, and not magnetic labeling of PSC-derived CMs, was used to separate the differentiated cells into non-CM and CM populations, to minimize manipulation of the CMs.

The sorted populations were plated as non-CMs, CMs, 1:9 co-cultures (1 part non-CM to 9 part CM), 3:7 co-cultures, 1:1 co-cultures, or 7:3 co-cultures at a density of 100,000 total cells per well to achieve monolayers. Co-cultures of hiPSC-CMs and non-CMs were visually confirmed throughout the culture period and at the experimental endpoints to have maintained monolayers of similar cell density (Supplementary Fig. S1).

Sorting was not performed for *GJA1* (encoding Cx43) knockdown (KD) experiments, but instead, hiPSC-CMs from highly efficient spinner culture differentiations (≥92% cTnI positive by flow cytometry) and non-CMs from cardiac differentiation wells with little-to-no beating were used (≤17% cTnI positive by flow cytometry, with the caveat that wells with no beating were prioritized for co-cultures versus flow cytometry characterization, so experimental non-CM samples were likely purer populations).

Cell populations were replated in B-27-supplemented RPMI-1640 media plus 20% FBS and 10 μM ROCK inhibitor on fibronectin-coated plates or Nunc Lab-Tek eight-well chamber slides (ThermoFisher) for further analysis. Nunc optical-bottom 96-well black-walled plates were used for ArcLight analysis. Media were changed to fresh B-27-supplemented media the day after plating.

For *GJA1* KD experiments, two different co-culture approaches were taken for each experiment. Either both the non-CMs and spinner hiPSC-CMs were collected and plated down simultaneously in a 1:1 ratio (50,000 cells each for 100,000 total cells plated in co-cultures, as before) or the non-CMs were plated on top of the spinner hiPSC-CMs, equal in number to the hiPSC-CMs that were originally plated per well (100,000 to achieve a monolayer). Both approaches led to successful co-culture formation and maintenance, so ArcLight data were collected from hiPSC-CMs within both types of co-cultures for each experiment.

Electrophysiological evaluation was performed between days 37 and 40 for the hiPSC-CMs in the *GJA1* KD experiments (17–20 days post-thaw of D20 cryopreserved hiPSC-CMs; 13–16 days after the combination of day 10 non-CMs; and day 24 spinner hiPSC-CMs in co-cultures).

For conditioned medium experiments, media were transferred to sorted CMs daily from wells of the pertinent cell population or co-culture condition, starting at 2 days postsort.

### ArcLight imaging and analysis

All ArcLight imaging was performed in a live cell incubation chamber at 5% CO_2_ and 37°C, with the media exchanged for Tyrode's solution (Sigma-Aldrich) before data acquisition. Optical APs were recorded from spontaneously beating cells using a Nikon Eclipse Ti microscope and NIS-Elements imaging software to measure GFP signal at 40× magnification and 50 frames per second.

A custom MATLAB program (Mathworks) was used to analyze AP parameters from the recorded data. Fluorescence intensity of the cells was corrected for background fluorescence. Then, following subtraction of fluorophore bleaching, the negative change in fluorescence from baseline over fluorescence at baseline (-ΔF/F) was calculated.

APD_50_ (action potential duration at 50% repolarization) was calculated as the width of the AP at 50% its height. Action potential duration at 90% repolarization (APD_90_) was determined as the time interval between when the signal reached 50% of maximum depolarization and 90% repolarization. Maximum upstroke velocity (V_max_) was calculated as the maximum instantaneous slope of a curve fit to the upstroke between 10% depolarization and maximum depolarization. AP amplitude was defined as the height of the AP at its maximum depolarization. AP interval was calculated as time between those AP maximums.

### Statistical analysis

Statistical analyses for each experiment are outlined in the figure legends. Data were expressed as either mean ± standard error of the mean or median ± interquartile range. Normality was determined using the D'Agostino-Pearson omnibus K2 test in GraphPad Prism. Either ordinary one-way analysis of variance or Kruskal-Wallis test was used for data sets with >2 groups being compared, followed by either two-tailed Student's *t*-test or two-tailed Mann-Whitney U test with Bonferroni correction. *P* < 0.05 was considered to be significant, with the alpha level for each comparison adjusted by Bonferroni correction in the case of multiple pairwise comparisons within a figure panel.

## Results

### Cardiac differentiation of hiPSCs produces distinct subpopulations of CMs and non-CMs

hiPSCs were reprogrammed from dermal fibroblasts (dFBs) derived from healthy individuals and subsequently differentiated to CMs by a monolayer-based directed differentiation protocol. Beating CMs were typically first observed between days 7 and 9 of differentiation, after which ArcLight was introduced by lentiviral transduction for all experiments involving electrophysiological evaluation. The utility of ArcLight for rapidly evaluating electrophysiological properties of human pluripotent stem cell-derived CMs has previously been demonstrated, including good correlation with patch-clamp recordings [14–16]. Importantly, this approach was chosen to allow noninvasive analysis of individual hiPSC-CMs within intact co-cultures.

**FIG. 1.**
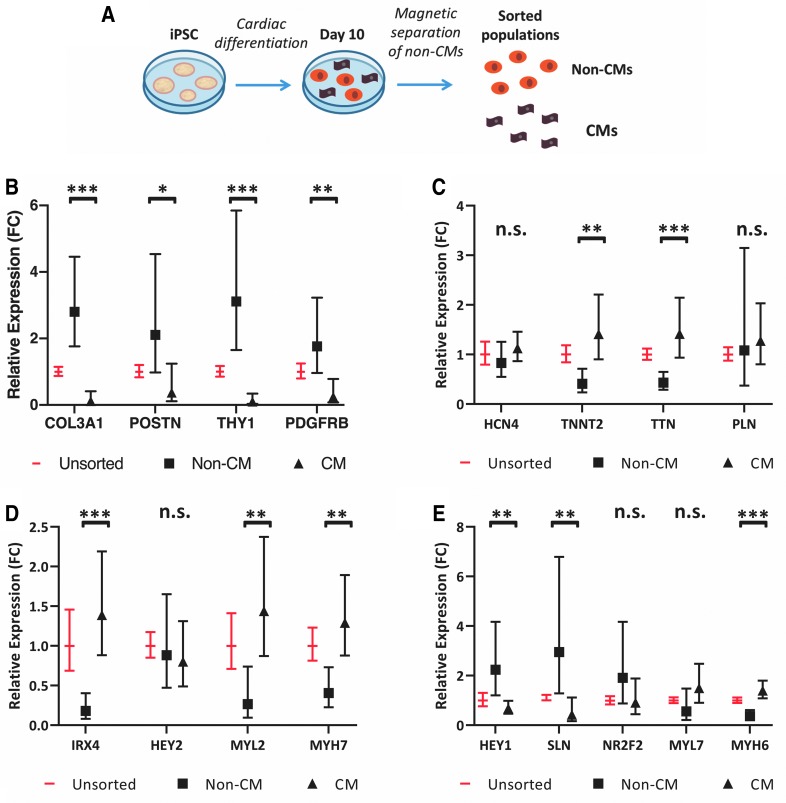

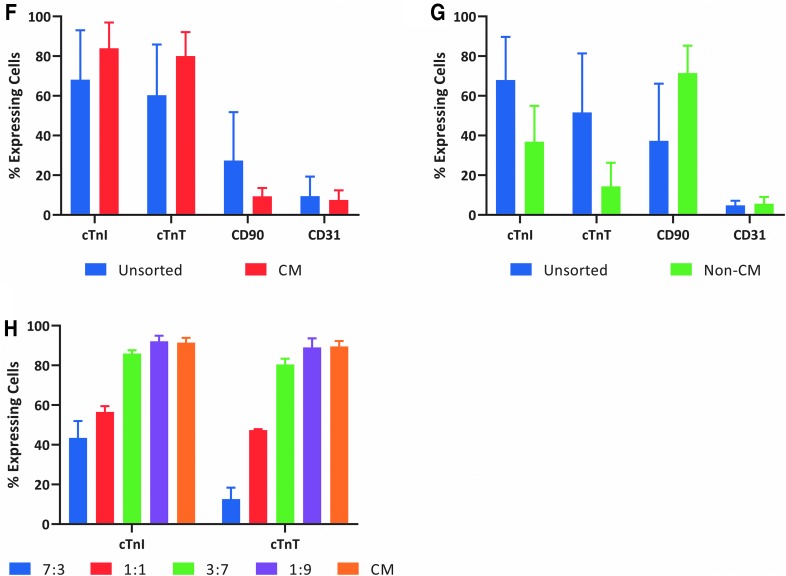
Characterization of sorted populations from cardiac differentiations reveals CM and cardiac fibroblast-like phenotypes. **(A)** Schematic of experimental design. Cardiac differentiations from hiPSCs were sorted on day 10 of differentiation into CM and non-CM populations. **(B–E)** Relative expression (FC) of cardiac fibroblast **(B)**, CM **(C)**, and ventricular CM **(D)** and atrial CM **(E)** genes in unsorted, non-CM, and CM subpopulations on day 10, as analyzed by qRT-PCR. Error bars represent range of fold change, calculated from standard deviation of ΔΔCt. Data are from eight sorts representing two clones each from three unrelated individuals. *P* values for non-CM versus CM samples were calculated from ΔΔCt values by a Student's *t*-test (*P ≤ 0.01; **P ≤ 0.001; ***P ≤ 0.0001). **(F)** Percentage of live cells in unsorted and CM populations positive for cTnI, cTnT, CD90, or CD31 by flow cytometry. Data are from 14 sorts representing 1–3 clones each from 4 unrelated individuals, analyzed between days 20 and 26. **(G)** Percentage of live cells in unsorted and non-CM populations positive for cTnI, cTnT, CD90, or CD31. Data are from nine sorts representing one to two clones each from three unrelated individuals, analyzed between days 20 and 26. **(H)** Percentage of live cells positive for cTnI or cTnT in co-culture conditions: 7 parts non-CM to 3 parts CM (7:3), 1:1, 3:7, 1:9, and CM only. Data are from four sorts representing two clones each from two unrelated individuals, analyzed on day 40. Data are represented as mean ± SEM. CM, cardiomyocyte; FC, fold change; hiPSCs, human induced pluripotent stem cells; non-CM, non-cardiomyocyte; n.s., not significant; qRT-PCR, quantitative reverse transcription–polymerase chain reaction; SEM, standard error of the mean.

A magnetic-activated cell sorting (MACS) approach was applied on day 10 to isolate non-CMs from the cardiac differentiations, producing putative non-CM and CM fractions (Fig. 1A). Day 10 was chosen to perform the sorting because beating CMs were already present, yet this time point was early enough to allow co-cultures to be established before notable maturation would occur [16–19].

To characterize the non-CM and CM fractions, gene expression was evaluated by quantitative reverse transcription–polymerase chain reaction (qRT-PCR) (Supplementary Table S1) in the two populations immediately following the day 10 sort (Fig. 1B–E). Previously, single-cell RNA-sequencing results from our laboratory demonstrated robust expression of numerous cardiac fibroblast-associated genes in non-CMs derived from a cardiac differentiation of hiPSCs [16]. Therefore, we focused specifically on several of these cardiac fibroblast genes for the qRT-PCR analysis.

Even as early as day 10, the non-CM population had higher expression of these genes compared to the CM population. Conversely, the CM fraction had more robust expression of several CM genes, particularly those associated with the contractile apparatus (including *TNNT2*, *TTN*, *MYL2*, *MYH7*, and *MYH6*). Interestingly, this was not the case for all the examined CM genes, including some transcription factors.

We also analyzed these populations by flow cytometry (Fig. 1F–H and Supplementary Fig. S2). Since we were unable to robustly detect the cell type markers by flow analysis on day 10, we replated the unsorted and the two sorted populations and collected them again on day 20 or later for analysis. When the CM and non-CM fractions were plated down separately, the non-CM populations had little-to-no beating and distinct morphology compared to the CM populations.

On average, over 80% of the cells in the CM populations (Fig. 1F) were positive for CM markers (cTnI and cTnT). While the study of cardiac fibroblasts has been traditionally hindered by the lack of a specific marker [20,21], CD90 has previously been used to sort cardiac fibroblast-like non-CMs from differentiated hiPSCs [11]. We found that about 10% of cells in the CM populations expressed the cardiac fibroblast marker CD90 (encoded by *THY1*), and a similar percentage expressed the endothelial marker CD31.

In the non-CM-sorted populations (Fig. 1G), on average, over 70% of the cells were positive for CD90. Interestingly, there was no enrichment for CD31, indicating that the non-CM and CM fractions were primarily distinguished by the proportion of CM and cardiac fibroblast-like cells.

Expression of cTnI and cTnT was assessed again on day 40 of differentiation in the CM population and in four co-culture conditions established on day 10: 7:3, 1:1, 3:7, and 1:9, non-CMs to CMs, respectively (Fig. 1H). Altogether, these evaluations showed an increasing percentage of cardiac troponin-positive cells in the co-cultures with a higher proportion of input CMs, with approximately half of the cells in the 1:1 co-cultures staining positive for cTnI or cTnT, as expected.

Importantly, this demonstrated that these variable distribution patterns could be retained until day 40. This was not unexpected since our group has previously found that proliferation rates for both CMs and non-CMs are very low by day 20 (<5%, unpublished data). Furthermore, a high percentage of cells (∼90%) in the CM fraction expressed cardiac troponin at this time point.

### Distinct influence of hiPSC-derived non-CMs upon CM electrophysiology requires physical interaction in co-cultures

**FIG. 2.**
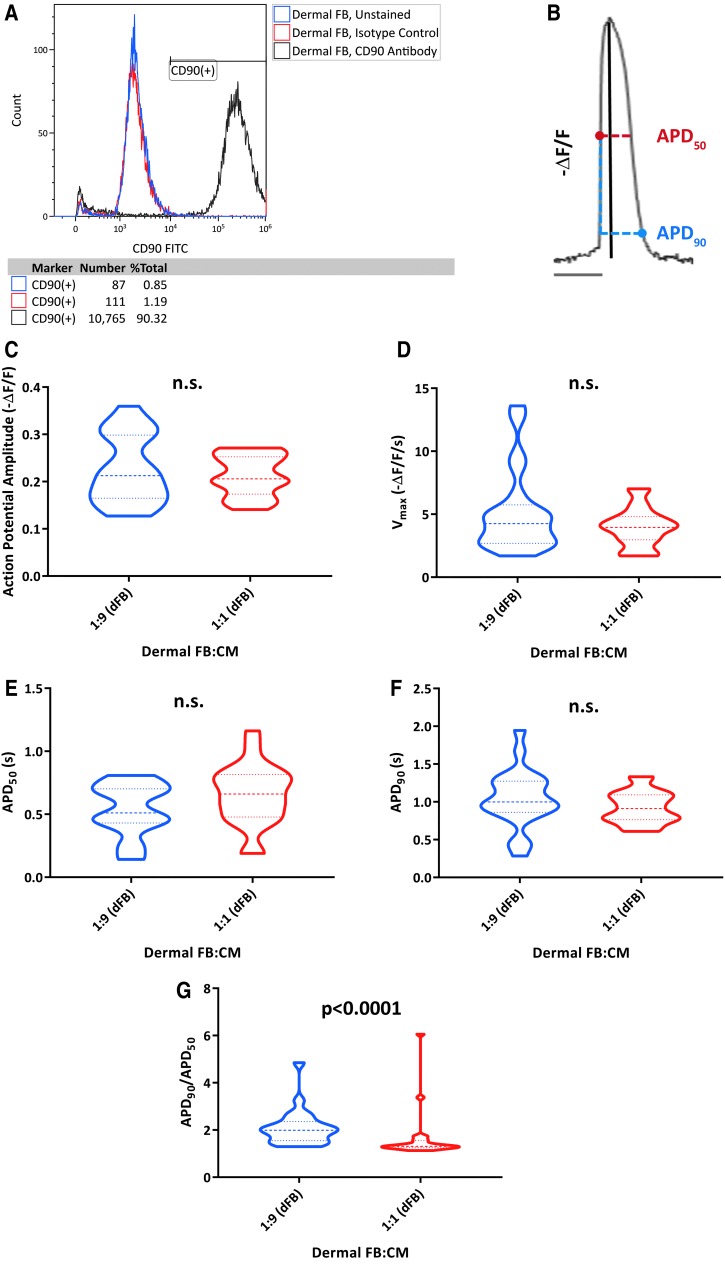
dFBs demonstrate the influence of a CD90-positive cell type upon hiPSC-CM electrophysiology. **(A)** Analysis of CD90 expression in dFBs by flow cytometry. **(B)** Optical tracings of negative change in fluorescence over fluorescence (-ΔF/F) permit analysis of AP properties of CMs, including APD_50_ (AP duration at 50% repolarization, *horizontal red dotted line*), APD_90_ (*horizontal blue dotted line*), amplitude (*black line*), and maximum upstroke velocity (V_max_, not shown). **(C–G)** Analysis of AP amplitude **(C)**, V_max_ **(D)**, APD_50_ **(E)**, APD_90_ **(F)**, and APD_90_/APD_50_ **(G)** for 1:9 (1 part dFB to 9 parts CM) and 1:1 co-cultures. Data were collected from two different hiPSC-CM clones on day 40 of differentiation. 1:9: *n* = 22 cells; 7:3: *n* = 22 cells. *Heavier dashed lines* within each violin indicate medians and *lighter dashed lines* indicate interquartile range. *P* values were calculated by either Student's *t*-test (AP amplitude, APD_50_, APD_90_) or Mann-Whitney U test (V_max_, APD_90_/APD_50_). AP, action potential; APD_50_, action potential duration at 50% repolarization; APD_90_, Action potential duration at 90% repolarization; dFBs, dermal fibroblasts; hiPSC-CM, human induced pluripotent stem cell-derived cardiomyocyte.

Before examining the effects of co-culturing non-CM and CM populations together, we first wanted to establish a baseline for the influence of a nonexcitable cell type on hiPSC-CM electrophysiology. We chose to evaluate co-cultures with dFBs, given the fibroblast-like phenotype of the hiPSC-derived non-CMs. Similar to the non-CMs and as expected, dFBs exhibited robust expression of CD90 (Fig. 2A). We co-cultured CMs sorted on day 10 of differentiation with dFBs derived from the same individual at ratios of 1:9 and 1:1 (dFB:CM), and then evaluated electrophysiology by ArcLight (Supplementary Fig. S3 and Supplementary Table S2) on day 40.

Altogether, we analyzed five different AP properties (Fig. 2B) by ArcLight: AP amplitude (Fig. 2C), maximum upstroke velocity (Fig. 2D), action potential duration at 50% or 90% repolarization (APD_50_, APD_90_) (Fig. 2E, F), and the APD_90_/APD_50_ ratio (Fig. 2G). Previously, it has been shown that ventricular-like APs can be characterized by an APD_90_/APD_50_ value of <1.4 [18], and thus, a more rectangular-like AP morphology. Moreover, our laboratory has previously shown that cells exhibiting an APD_90_/APD_50_ value of <1.4, as measured by ArcLight, do not demonstrate a profound response to pharmacological inhibition of the atrial ion current I_Kur_, supportive of a more ventricular-like electrophysiological phenotype [16].

The only parameter that was significantly different between the two conditions was decreased APD_90_/APD_50_ with a higher proportion of dFBs. However, AP duration and shape were difficult to accurately assess due to the unusually high propensity for arrhythmias (18% of cells analyzed for both conditions exhibited early and/or delayed after depolarizations) in these co-cultures.

**FIG. 3.**
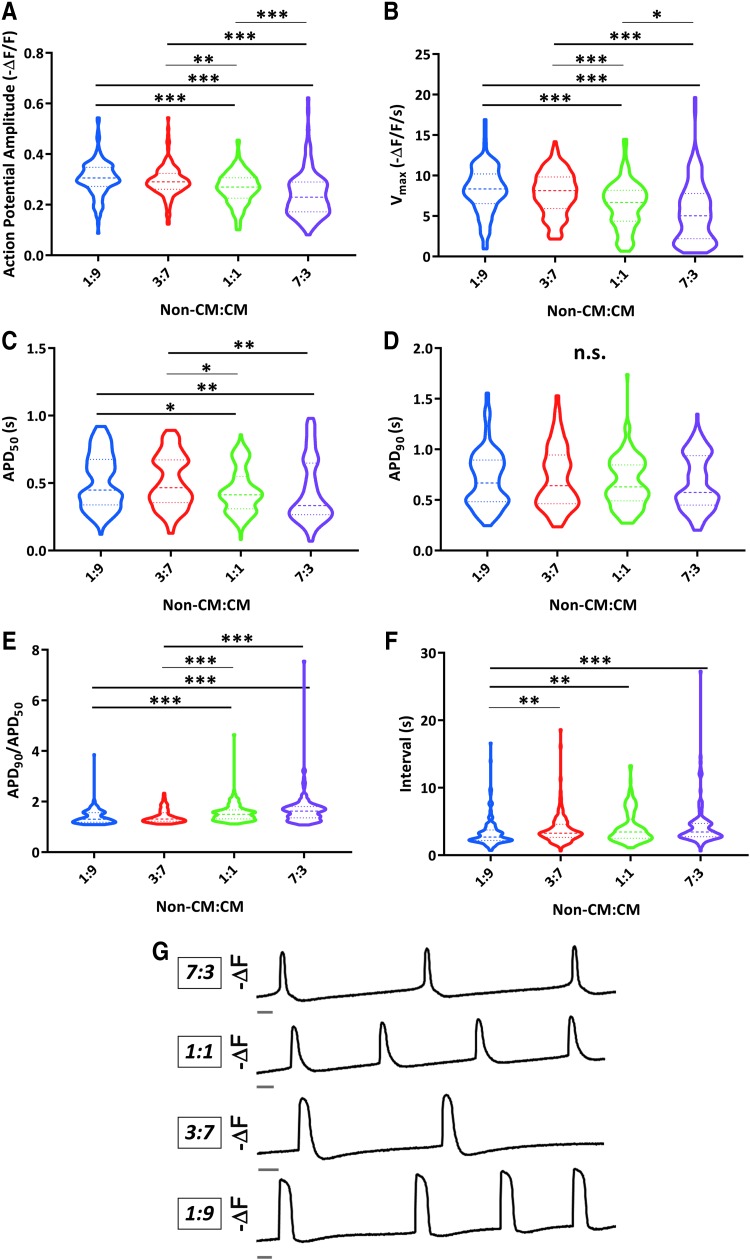
Varied electrophysiological properties of hiPSC-CMs due to differing ratios of non-CMs versus CMs in co-cultures. **(A–F)** Analysis of optical AP amplitude **(A)**, maximum upstroke velocities (V_max_) **(B)**, APD_50_ (AP duration at 50% repolarization) **(C)**, APD_90_ **(D)**, APD_90_/APD_50_ **(E)**, and interval between APs **(F)** for co-cultures with 1 part non-CMs to 9 parts CMs 1:9, 3:7 co-cultures, 1:1 co-cultures, and 7:3 co-cultures. Data were collected from eight independent differentiations representing seven clones from three unrelated individuals between days 39 and 40 of differentiation; 1:9: *n* = 152 cells; 3:7: *n* = 149 cells; 1:1: *n* = 160 cells; and 7:3: *n* = 138 cells. *Heavier dashed lines* within each violin indicate medians and *lighter dashed lines* indicate interquartile range. Data were initially analyzed by a Kruskal-Wallis test (n.s. for APD_90_ and *P* < 0.0001 for all other parameters) and pairwise *P* values were calculated by Mann-Whitney U test for AP amplitude, V_max_, APD_50_, APD_90_/APD_50_, and interval (*P ≤ 0.0083; **P ≤ 0.001; ***P ≤ 0.0001). **(G)** Representative inverted fluorescence traces (-ΔF) of optical APs for 7:3, 1:1, 3:7, and 1:9 co-culture conditions from the same experiment. Trace bars indicate 1 s.

Interestingly, when we applied the same analysis approach to hiPSC-CMs co-cultured with hiPSC-derived non-CMs, we observed distinct phenotypes compared to the dFB co-cultures. Namely, we saw higher AP amplitude (Fig. 3A) and V_max_ (Fig. 3B) in the 1:9 and 3:7 co-cultures, which had the greatest percentage of CMs. These phenotypes are hallmarks of enhanced cardiac electrophysiological maturation [6]. Increasing the proportion of CMs also increased APD_50_ values (Fig. 3C). However, there were no significant differences in APD_90_ between the conditions (Fig. 3D). Consequently, APD_90_/APD_50_ values were lower for the co-cultures conditions with higher percentage of CMs (Fig. 3E), with this shift being in the opposite direction to what we had previously seen for the co-cultures with a higher proportion of CMs versus dFBs.

These phenotypes did not appear to be clearly linked to differences in beating frequencies, as there was no significant difference in the interval between APs for 3:7, 1:1, or 7:3 co-culture conditions, with hiPSC-CMs in the 1:9 condition actually exhibiting slightly shorter intervals despite longer APD_50_ values (Fig. 3F). Altogether, this suggested that contamination with non-CMs has a distinctive influence upon hiPSC-CMs, with sufficiently abundant quantities having the ability to hamper properties associated with cardiac electrophysiological maturation and the development of ventricular-like AP morphologies (Fig. 3G).

To gain more insight into phenotypic differences between the co-cultures, we also compared expression of several atrial (*MYL7* and *KCNA5*) or ventricular (*MYL2*) genes in CM populations, which were sorted back out of co-cultures on day 40 of differentiation (Supplementary Fig. S4A–C). We observed lower *MYL2* expression in hiPSC-CMs from the 7:3 condition compared to ratios 3:7 and 1:9, and CM (no co-culture) conditions, further implying decreased ventricular maturation for hiPSC-CMs cultured with abundant non-CMs.

We did not detect any significant difference in structural features such as number of nuclei per CM (Supplementary Fig. S4D) or CM size (Supplementary Fig. S4E) between the four co-culture conditions, suggesting that only a subset of maturation-associated properties is altered by non-CM content of the culture environment.

We next wanted to evaluate whether the impact of non-CMs upon hiPSC-CM electrophysiological development could be attributed to paracrine effects. To do so, we transferred fresh conditioned media daily to sorted CM populations, starting on day 12 (2 days postsort) until approximately day 40 of differentiation. This conditioned medium came from one of four different conditions: sorted CMs, sorted non-CMs, 1:9 co-cultures, or 7:3 co-cultures.

**FIG. 4.**
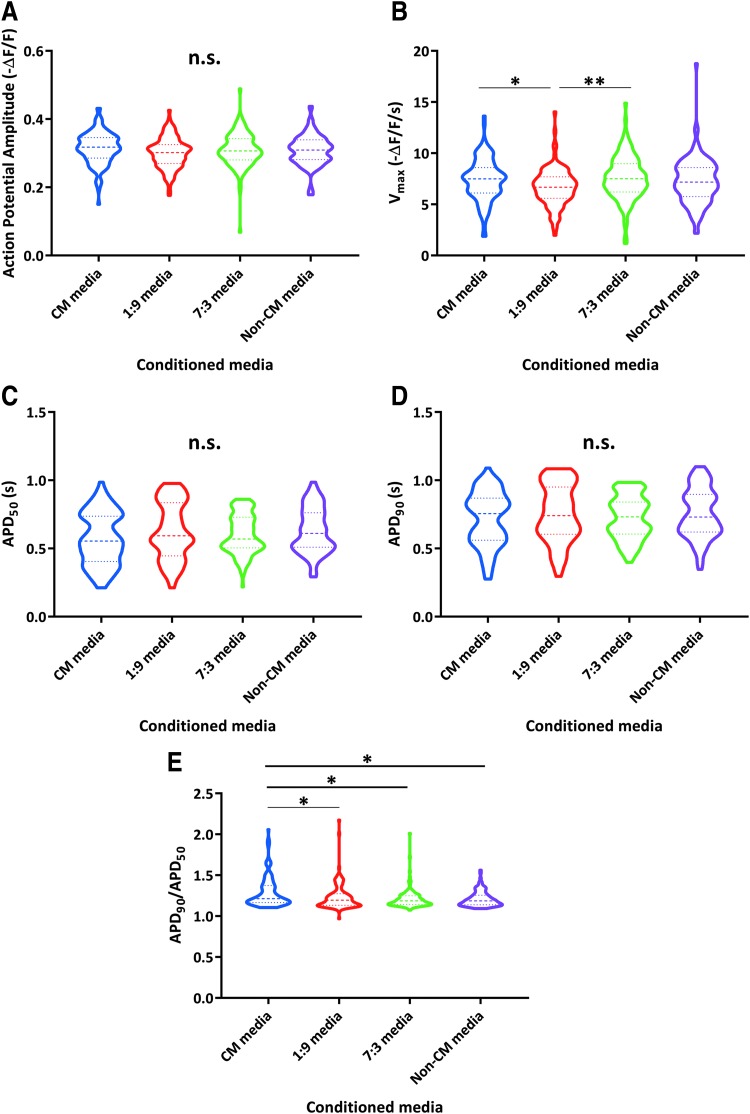
Non-CMs do not have a notable influence on CM electrophysiology through paracrine effects. **(A-E)** Analysis of optical AP amplitude **(A)**, maximum upstroke velocities (V_max_) **(B)**, APD_50_ (AP duration at 50% repolarization) **(C)**, APD_90_ **(D)**, and APD_90_/APD_50_ **(E)** for CMs cultured with conditioned media from CMs, non-CMs, co-culture with 1 part non-CM to 9 parts CM (1:9), or 7:3 co-culture. Data were collected from six independent differentiations representing five clones from two unrelated individuals between days 38 and 40 of differentiation. CM media: *n* = 121 cells; 1:9 media: *n* = 118 cells; and 7:3 media: *n* = 121 cells; non-CM media: *n* = 119 cells. *Heavier dashed lines* within each violin indicate medians and *lighter dashed lines* indicate interquartile range. Data were initially analyzed by a Kruskal-Wallis test (*P* < 0.01 for V_max_, *P* < 0.001 for APD_90_/APD_50_, and n.s. for all others) and pairwise *P* values were calculated by Mann-Whitney U tests for V_max_ and APD_90_/APD_50_ (*P ≤ 0.0083; **P ≤ 0.001).

When we evaluated the cells cultured with CM or non-CM-conditioned media at an intermediate time point (approximately day 25), there were no significant differences between the treatments for any of the parameters (Supplementary Fig. S5). When we assayed the cells again 2 weeks later (approximately day 40), we still found few electrophysiological differences between CMs cultured with the various conditioned media (Fig. 4). The most notable finding was slightly increased APD_90_/APD_50_ values after treatment with CM-conditioned media, but this was not consistent in directionality with the phenotype we initially observed for co-culture with non-CMs, and 80% (97/121) of the analyzed cells still had ratios of <1.4.

Therefore, we concluded that paracrine effects were likely not the primary mechanism underlying the phenotypes we had initially observed for the co-cultures with non-CMs.

### The role of Cx43 in mediating interactions between non-CMs and hiPSC-CMs

Since the hiPSC-derived non-CMs appeared to have a distinct influence upon hiPSC-CM electrophysiology in co-culture conditions, and the resulting phenotypes did not appear to be mediated through paracrine signaling, we next examined the possible role of cell-cell coupling. Cx43 has been reported to be the primary connexin involved in mediating gap junction coupling between tissue-derived ventricular CMs and cardiac fibroblasts [21].

**FIG. 5.**
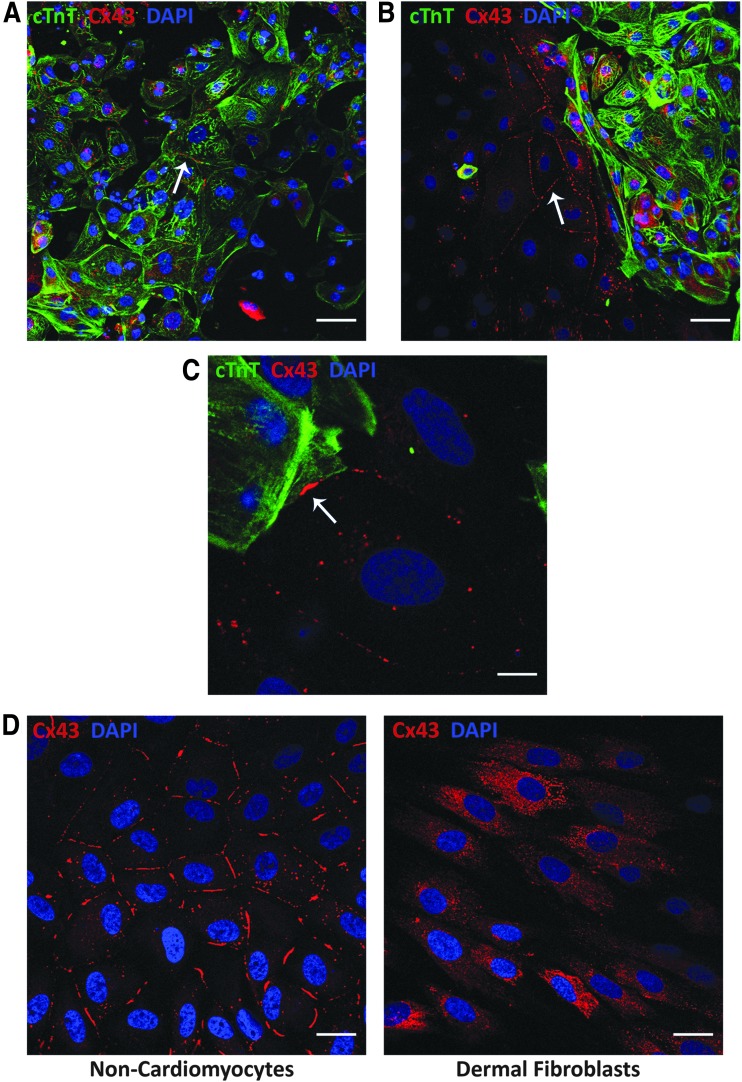
Both hiPSC-CMs and non-CMs, but not dFBs, exhibit Cx43 expression at cell-cell interfaces. **(A**, **B)** Immunofluorescence of cardiac troponin T (cTnT, *green*) and Cx43 (*red*) in hiPSC-CMs and non-CMs on day 41 of differentiation, showing Cx43 expression between cTnT-positive CMs **(A)** and between cTnT-negative non-CMs **(B)**. Scale bars represent 50 μM. **(C)** Immunofluorescence of Cx43 expression at the interface of a cTnT-positive CM and a non-CM on day 41. Scale bars represent 10 μM. A-C are representative of four independent differentiations of three different clones representing two unrelated individuals between days 38 and 41. *White arrows* indicate examples of Cx43 staining at cell-cell boundaries. **(D)** Immunofluorescence of Cx43 in day 38 non-CMs (*left*) and dFBs (*right*, representative of cells from three different individuals) derived from the same individual. Scale bars represent 50 μM. Cx43, connexin 43.

Therefore, we performed immunofluorescence staining to evaluate whether the non-CMs express Cx43, and in which regions of the cell it localizes. Not surprisingly, we were able to see punctate staining for Cx43 at the interface between cTnT-positive CMs (Fig. 5A). Interestingly, we also observed a similar phenomenon at the interfaces between non-CMs (Fig. 5B). This was consistent with the detection of *GJA1* (encodes for Cx43) gene expression in both CMs and non-CMs from our previously published single-cell RNA-sequencing study [16]. Furthermore, we also observed Cx43 expression at the interface between some CMs and non-CMs, suggesting that these different cell types might be able to couple through gap junctions (Fig. 5C).

When we compared the subcellular localization of Cx43 for hiPSC-derived non-CMs and dFBs, we observed that only the non-CMs demonstrated notable Cx43 expression at cell-cell interfaces (Fig. 5D), potentially contributing to the distinct electrophysiological influence of the non-CMs compared to the dFBs.

To study the impact of these Cx43-mediated cell-cell connections upon hiPSC-CM electrophysiology, we knocked down *GJA1* in cardiac differentiations by short hairpin RNAs (shRNAs). We examined expression of Cx43 by immunofluorescence in differentiations with a high proportion of non-CMs, to ensure that the KD was effective in our target cell type at the protein level.

**FIG. 6.**
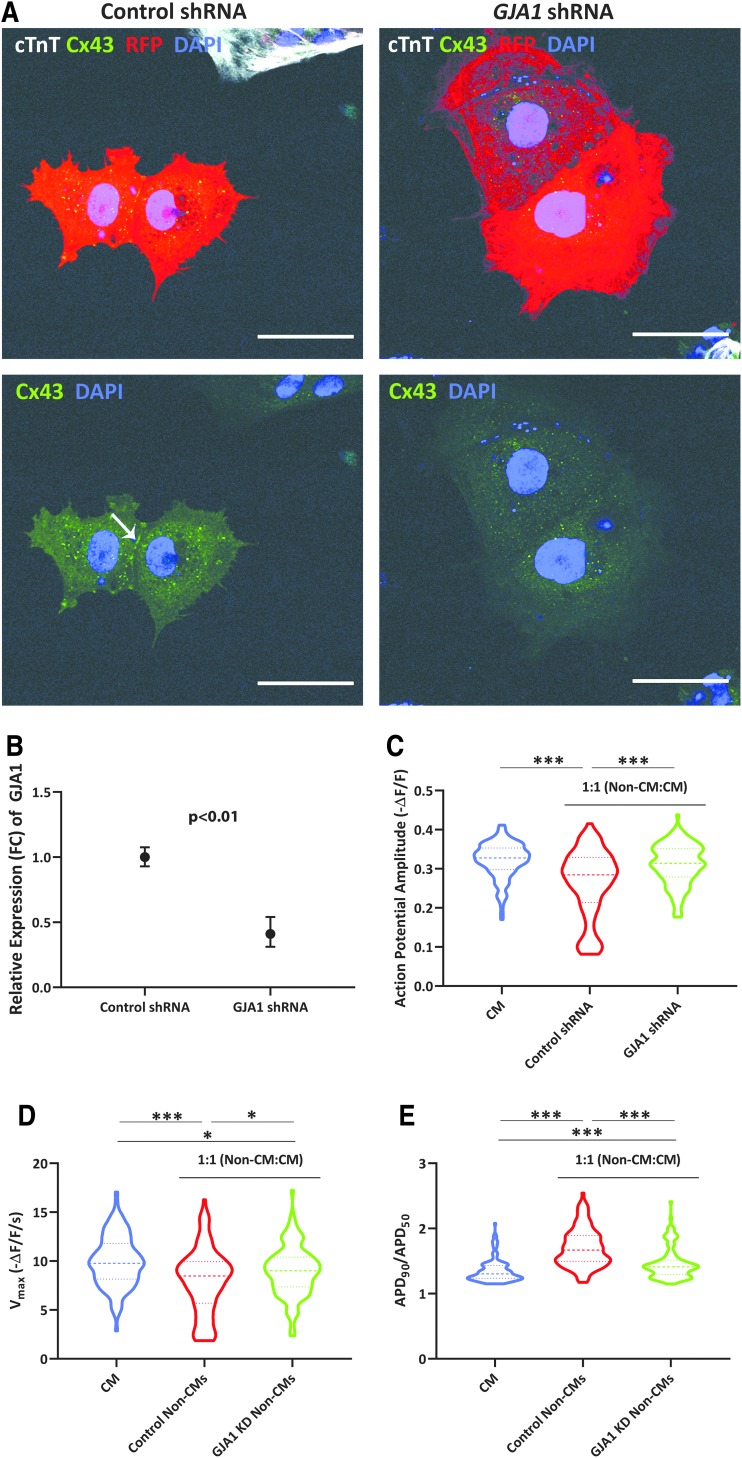
Non-CMs mediate CM electrophysiology in a Cx43-dependent manner. **(A)** Immunofluorescence of Cx43 (*green*) in non-CMs transduced with either control or *GJA1*-targeted shRNAs, as indicated by RFP expression. Most stained cells did not express cTnT (*white*). Images are representative of two independent experiments. *White arrow* indicates Cx43 staining at cell-cell boundaries. Scale bars represent 50 μM. **(B)** Relative expression (FC) of *GJA1* for knockdown and control samples, as analyzed by qRT-PCR. Error bars represent range of fold change, calculated from standard deviation of ΔΔCt. Data are from three differentiations representing clones from two unrelated individuals. *P* value was calculated from ΔΔCt values by a Student's *t*-test. **(C–E)** Analysis of optical AP amplitude **(C)**, maximum upstroke velocities (V_max_) **(D)**, and APD_90_/APD_50_ **(E)** for CMs and 1:1 co-cultures of CMs and control or *GJA1* knockdown non-CMs. Data were collected in three independent experiments involving two clones in total (from distinct individuals) for both non-CMs and CMs. CMs: *n* = 120 cells (60 plated together and 60 non-CMs plated on top); control shRNA: *n* = 116 cells (60 plated together and 56 non-CMs plated on top); and *GJA1* shRNA: *n* = 120 cells (60 plated together and 60 non-CMs plated on top). *Heavier dashed lines* within each violin indicate medians and *lighter dashed lines* indicate interquartile range. Data were initially analyzed by ANOVA (V_max_; *P* < 0.0001) or a Kruskal-Wallis test (AP amplitude, APD_90_/APD_50_; *P* < 0.0001) and pairwise *P* values were calculated by either a Student's *t*-test (V_max_) or Mann-Whitney U test (AP amplitude, APD_90_/APD_50_). *P ≤ 0.0167; ***P ≤ 0.0001. ANOVA, analysis of variance; shRNA, short hairpin RNA.

While Cx43 expression was not as robust as we had seen on approximately day 40 of differentiation, we still observed a higher prevalence of Cx43 localized at cell-cell interfaces for control versus *GJA1* KD non-CMs on day 17 (10 days post-transduction). This indicated that we were able to modulate Cx43 expression in the non-CMs, including at cell-cell boundaries (Fig. 6A). cTnT(+) CMs were uncommon, were consistent with flow cytometry characterization, and had comparatively low expression of RFP [∼20% of observed cTnT(+) cells were also RFP(+)]. Quantitatively, we also detected a significant decrease in the expression of *GJA1* in KD versus control shRNA-treated cells by qRT-PCR (Fig. 6B), including for differentiations that were primarily composed of non-CMs.

For the functional assessments, we also utilized *GJA1* KD cellular populations, which were primarily composed of non-CMs, and co-cultured those cells with high-purity ArcLight-transduced spinner culture-derived hiPSC-CMs for ∼2 weeks. Overall, this approach allowed for high purities of both CMs and non-CMs, and thus enabled modulation of Cx43 in the non-CMs, while minimizing impact upon the CM subpopulation in the co-cultures.

The co-culture period started on day 10 of differentiation for the non-CMs, as in the previous experiments, and on day 24 (4 days post-thaw) for the CMs. Once again, we maintained these co-cultures until the hiPSC-CMs reached ∼40 days postdifferentiation. We verified co-culture formation at the experimental endpoints by fluorescent imaging of the RFP-expressing non-CM fraction (representing transduction with either *GJA1*-targeted or control shRNAs) and the ArcLight-expressing CM fraction, which showed an intermingling of the two cell populations across the wells (Supplementary Fig. S6). We also confirmed that CMs in co-cultures had a similar beating rate when plated down together with either *GJA1* KD or control non-CMs (Supplementary Fig. S7).

We then compared electrophysiological parameters for three different conditions: co-cultures with *GJA1* KD non-CMs, co-cultures with control shRNA-transduced non-CMs, and CM cultures (no added non-CMs). Consistent with what we had seen previously, the CMs in the co-cultures with control non-CMs exhibited lower AP amplitude (Fig. 6C) and V_max_ (Fig. 6D) and higher APD_90_/APD_50_ (Fig. 6E) compared to the CM cultures. Importantly, since only the CM populations were transduced with ArcLight, we could attribute these phenotypes to electrophysiological differences in the CMs themselves rather than the detection of APs in the non-CMs. Notably, for the CMs co-cultured with *GJA1* KD non-CMs, AP amplitude, V_max_, and APD_90_/APD_50_ were shifted toward values more similar to those of the CM cultures.

Overall, these findings suggest that a decreased *GJA1* (and subsequently Cx43) expression, specifically in non-CMs, is sufficient to diminish the impact of non-CMs upon cardiac electrophysiology.

## Discussion

The precise identity and functional implications of the hiPSC-derived non-CMs produced in the cardiac differentiation process continue to merit ongoing consideration. CD90 has previously been shown to label the majority of non-CMs across different hiPSC lines. These cells have also been found to robustly express several fibroblast markers in common with CD90^+^ non-CMs isolated from heart tissue. Furthermore, hiPSC-derived CD90^+^ non-CMs have been shown to exhibit low expression of pluripotency and endothelial markers [11]. Other studies have similarly described the non-CMs as having a myofibroblast-like phenotype [13].

Previous work in our laboratory demonstrated that by day 40 of differentiation, hiPSC-derived non-CMs exhibited robust expression of numerous cardiac fibroblast-associated genes and were characterized by extracellular matrix-related gene functions [16]. Another recent single-cell RNA-sequencing analysis likewise revealed that the non-CM population is enriched for fibroblast-like cells marked by *THY1* (encodes for CD90) and exhibits higher expression of genes associated with extracellular matrix deposition, motility, and cell adhesion. Interestingly, those cells were also found to have enriched expression of genes associated with outflow tract development, suggesting that the non-CMs may be cardiac outflow tract-like cells. However, the authors posited that further studies are still needed to fully elucidate the identity of these noncontractile cell types [22].

Herein, we additionally found that the non-CMs and hiPSC-CMs demonstrate robust expression of several cardiac genes. It has previously been reported that tissue-derived cardiac fibroblasts highly express some genes typically associated with CM development and function [23]. In fact, murine cardiac fibroblasts are transcriptionally more similar to CMs than to tail-derived fibroblasts [24].

This study reports that non-CMs produced in the cardiac differentiation of hiPSCs influence electrophysiological phenotypes of hiPSC-CMs. We chose to use the genetically encoded voltage indicator ArcLight to analyze AP properties since this is a noninvasive, nontoxic approach that allowed high-throughput characterization of hiPSC-CMs within intact co-cultures [14,15]. In addition, ArcLight measurements have been shown to correlate closely to simultaneous patch-clamp recordings [15].

Our group has previously found ArcLight and patch clamp to be comparable in their ability to phenotype CM subtype-like AP morphologies. Furthermore, we have utilized ArcLight to detect maturation-associated electrophysiological changes over a time course of transcriptional maturation. Throughout the current and prior studies, we have successfully introduced ArcLight to CMs produced by multiple differentiation protocols between 1 and 6 weeks of differentiation [16]. However, since this is an optically based approach, it does not allow measurement of absolute membrane potentials, and thus cannot be used to evaluate maximum diastolic potential. Therefore, obtaining direct AP recordings of hiPSC-CMs within co-cultures for follow-up studies will also be of value.

Ultimately, we found decreased AP amplitude and V_max_ by day 40 of differentiation for co-cultures with high non-CM content (∼50% or greater), compared to co-cultures with higher hiPSC-CM purity. We also observed less ventricular-like AP morphologies for the cultures with 50% or greater non-CMs. This indicates that the presence of excessive non-CMs can hamper properties associated with hiPSC-CM electrophysiological maturation. These results also suggest that interactions between hiPSC-CMs and non-CMs could perhaps contribute to the variability in electrophysiological properties of hiPSC-CMs, which has previously been reported [25]. Overall, we demonstrate the role of non-cell-autonomous external influences upon the functional properties of an hiPSC-derived cell type, which could additionally be applicable to other differentiation systems.

Interestingly, an early study examining the effects of non-CMs upon the electrophysiological maturation of human embryonic stem cell-derived CMs showed that differentiations purified to >95% CMs before day 20 of differentiation resulted in halted development of several ion channels as well as decreased AP amplitude and V_max_ by day 60. Reintroduction of non-CMs could rescue this stunted electrophysiological maturation [12].

However, a more recent report examined scaffold-free engineered cardiac tissues with 25%, 50%, 70%, or 90% hiPSC-CMs (plus non-CMs), and ultimately showed that rising AP slopes were highest for tissues containing 70% CMs. Other indications of enhanced maturation were also apparent for the 70% and/or 90% hiPSC-CM conditions [13]. In conjunction with our findings, this could suggest that having the correct balance of non-CMs (perhaps representing a measurable yet minor proportion) is critical for promoting optimal maturation.

It is possible that we were not able to achieve a high enough CM purity in our study to observe any indication of decreased maturation, as previously reported for cultures with >95% CMs. However, we achieved ∼90% CMs in the 1:9 condition on day 40, and ≥92% in the spinner culture-derived CMs, suggesting that only a fairly minor non-CM subpopulation may be necessary to provide a maturation benefit.

We also expanded upon these previous studies by delving further into the mechanisms underlying the interactions between hiPSC-CMs and non-CMs. Ultimately, we found that treatment with conditioned media from non-CMs did not appear to recapitulate our original findings of decreased AP amplitude and V_max_ plus increased APD_90_/APD_50_ with increased non-CM content. Although we could not discount other paracrine-mediated influences of non-CMs upon hiPSC-CMs, including a slight effect on APD_90_/APD_50_, this suggested that direct cell-cell contact was required for the particular phenotypes we observed in co-cultures.

Therefore, we investigated the potential role of connexin-mediated cell-cell interactions, on the basis of previous studies involving tissue-derived cardiac fibroblasts. Specifically, cardiac fibroblasts have been shown to express Cx40, Cx43, and Cx45, with Cx43 being the primary connexin found to couple cardiac fibroblasts to each other and to ventricular CMs when these tissue-derived cells are cultured in vitro [20,21,26–28]. It has even been demonstrated that non-CMs in murine myocardial scar tissues can electrically couple to CMs in vivo, mediated by Cx43 [29].

These connexin-mediated interactions may furthermore have functional implications. Mathematical models have proposed that cardiac fibroblasts can act as current sinks and ultimately decrease CM V_max_ and AP amplitude [21,30–32]. In vitro assays have similarly shown that Cx43-mediated coupling between neonatal rat CMs and myofibroblasts results in decreased CM V_max_ [33]. Moreover, computational models and in vitro systems have reported that cardiac fibroblasts have the ability to either increase or decrease CM APD, depending on factors such as the resting membrane potentials of the cardiac fibroblasts, density of cardiac fibroblasts, and degree of CM coupling [21,30–34]. Interestingly, human cardiac fibroblasts also influence the calcium handling properties of hiPSC-CMs through direct contact or close proximity [35].

We observed that the hiPSC-derived non-CMs express Cx43 at cell boundaries, including at interfaces with both hiPSC-CMs and other non-CMs. To evaluate the functional implications of non-CM Cx43 expression, we knocked down Cx43 (*GJA1*) in primarily non-CM populations and co-cultured either these KD or control non-CMs with ArcLight-transduced hiPSC-CMs.

We found that the co-cultures with control non-CMs exhibited distinct phenotypes compared to a purer CM population, which were consistent with what we had seen previously (namely lower AP amplitude, decreased V_max_, and increased APD_90_/APD_50_). Strikingly, KD of Cx43 resulted in electrophysiological phenotypes more similar to those of the hiPSC-CMs not in co-cultures. Ultimately, this indicates a role for Cx43 in mediating functionally relevant interactions between hiPSC-derived non-CMs and CMs.

dFBs have also been previously reported to express Cx43 [36,37], but we did not see the same expression pattern at cell-cell interfaces between dFBs as we had seen for the hiPSC-derived non-CMs. This difference in Cx43 localization could potentially contribute to the distinct effect of these different cell types upon hiPSC-CM electrophysiology. However, it is also possible that differences in proliferation rates and/or size between dFBs and non-CMs could have resulted in dissimilarities in cell density, and thus differentially impacted electrophysiological behavior.

Our results suggest that the electrophysiological influence of hiPSC-derived non-CMs may mirror the effects that tissue-derived cardiac fibroblasts have been predicted or reported to have. However, it should be noted that there is also a possibility of signaling molecules such as microRNAs (miRNAs) passing between non-CMs and CMs through gap junctions, and thus modulating CM functionality. As precedence for this, one study found that co-culture of mouse embryonic stem cell-derived CMs with rat endothelial cells enhanced CM maturation by upregulation of specific miRNAs. Moreover, the authors identified that only a lysate of endothelial cells and not endothelial cell conditioned media or endothelial-produced extracellular matrix enhanced sarcomeric organization of mouse embryonic stem cell-derived cardiomyocytes (ESC-CMs), perhaps indicating that these miRNAs were transferred across gap junctions [38].

It has also been observed that the distribution of hiPSC-CM AP morphologies differs for confluent monolayers versus sparsely seeded cells [39]. While we utilized monolayer co-cultures in our experiments and did not observe any clear indication of differences in cell density between the various non-CM co-culture conditions, there is potential that varying CM content could, in part, have a similar effect to modulating density. More detailed study of the mechanisms underlying the role of hiPSC-CM density in cardiac electrophysiology could help elucidate that possibility.

Along those lines, the geometry of hiPSC-CM cell patterning has been found to influence electrophysiological properties, such as susceptibility to arrhythmias [40]. In one study, it was seen that hiPSC-CMs grown in patterned islands of a larger size show increased maturation in terms of both electrophysiology and gene expression. This included greater AP amplitude and V_max_, although no difference in AP duration. The authors concluded that hiPSC-CMs cultured in those larger islands predominately mature by short-range interactions with neighboring CMs, although it was unclear whether those interactions were mechanical or biochemical in nature [41].

Therefore, it is possible that the functional effects of increased non-CM content that we saw could reflect a combination of both non-CM-dependent mechanisms and decreased direct CM-CM interactions.

It is important to note that depending on nature of the mechanisms underlying the observed phenomena, as discussed above, the phenotypes may represent the context-dependent nature of hiPSC-CM electrophysiological properties, rather than stable phenotypic changes. Further studies will be necessary to investigate the acute effects of altering non-CM content during the culture period, building upon prior findings that the electrophysiological effects of eliminating non-CMs on hiPSC-CM maturation can be reversed [12].

In addition, even interactions between CMs and non-CMs in tissues are still not fully understood, and many of the previous studies have been performed with non-human cells or in silico. If hiPSC-derived non-CMs have similar properties to cardiac fibroblasts, they could potentially be used to further model these cell-cell interactions using human cells, which would have implications for studying cardiac remodeling following myocardial infarction and arrhythmogenesis [21].

Finally, our studies demonstrate that not only can the presence of non-CMs impact phenotypes indicative of electrophysiological maturation but also the prevalence of APs with a more rectangular, ventricular-like morphology. This is particularly interesting in light of the observation by Iseoka et al. that engineered cardiac tissues with high CM purity (90%) exhibited the highest percentage of MLC2v^+^/MLC2a^−^ CMs [13], and our similar findings that hiPSC-CMs from the 7:3 co-cultures had the lowest *MYL2* expression. Further studies will be required to show whether CM purity affects any other ventricular-associated phenotype. It may also be informative to examine the role of non-CMs in other differentiation protocols designed to promote the development of atrial-like or nodal-like CMs.

Overall, our findings provide mechanistic insight into the active influence that non-CMs have upon maturation-associated electrophysiological properties of hiPSC-CMs and demonstrate that expression patterns of Cx43 in non-CMs alter the normal ventricular electrophysiology in neighboring CMs. While a common goal has often been to increase CM purity and thus effectively eliminate non-CMs [10], it is now also becoming apparent that the presence of some non-CMs could be desirable for some tissue engineering [13] or disease modeling purposes [11]. A more complete understanding of cell-cell interactions within the culture system can ultimately allow for more informed decisions regarding how to achieve the optimal cellular composition for the intended application.

More broadly, our results demonstrate the role of environmental influences, including supporting cell types, in modulating the functional properties of hiPSC-derived cells, which in particular have implications for the creation of purpose-built in vitro models and cell-based therapeutic products.

## Supplementary Material

Supplemental data
