# Supplementary material for: Human Induced Pluripotent Stem Cell-Derived Non-Cardiomyocytes Modulate Cardiac Electrophysiological Maturation Through Connexin 43-Mediated Cell-Cell Interactions
Source: Stem Cells Dev. 2020 Jan 10;29(2):75–89. doi: 10.1089/scd.2019.0098 (PMC6978788; doi:10.1089/scd.2019.0098)
Supplement: Supplemental data [file Supp_Data.pdf]

Supplementary Data

Supplementary Materials and Methods

Human induced pluripotent stem cell generation, culture, and characterization

Skin biopsies were obtained from de-identified healthy individuals in accordance with institutional regulations (Mayo Clinic IRB 10-006845, Clinical Trials Identifier NCT01860898). Human induced pluripotent stem cells (hiPSCs) were then generated from the primary fibroblasts derived from these biopsies by ReGen Theranostics (Rochester, MN). Sendai reprogramming and selection of clones were performed using CytoTune-iPS Sendai Reprogramming Kits (Invitrogen) according to the manufacturer's instructions. All hiPSC clones were cultured in mTeSER1 medium (STEMCELL Technologies) and passaged by either mechanical passaging or ReLeSR (STEMCELL Technologies).

The clones used were verified to have no known disease-causing mutations in mitochondrial DNA (mtDNA). Sequencing of mtDNA was performed by the Department of Laboratory Medicine and Pathology Molecular Genetics Laboratory and the Medical Genome Facility at Mayo Clinic. Analysis of mtDNA next-generation sequencing data was done using HaploGrep software and the MITOMAP

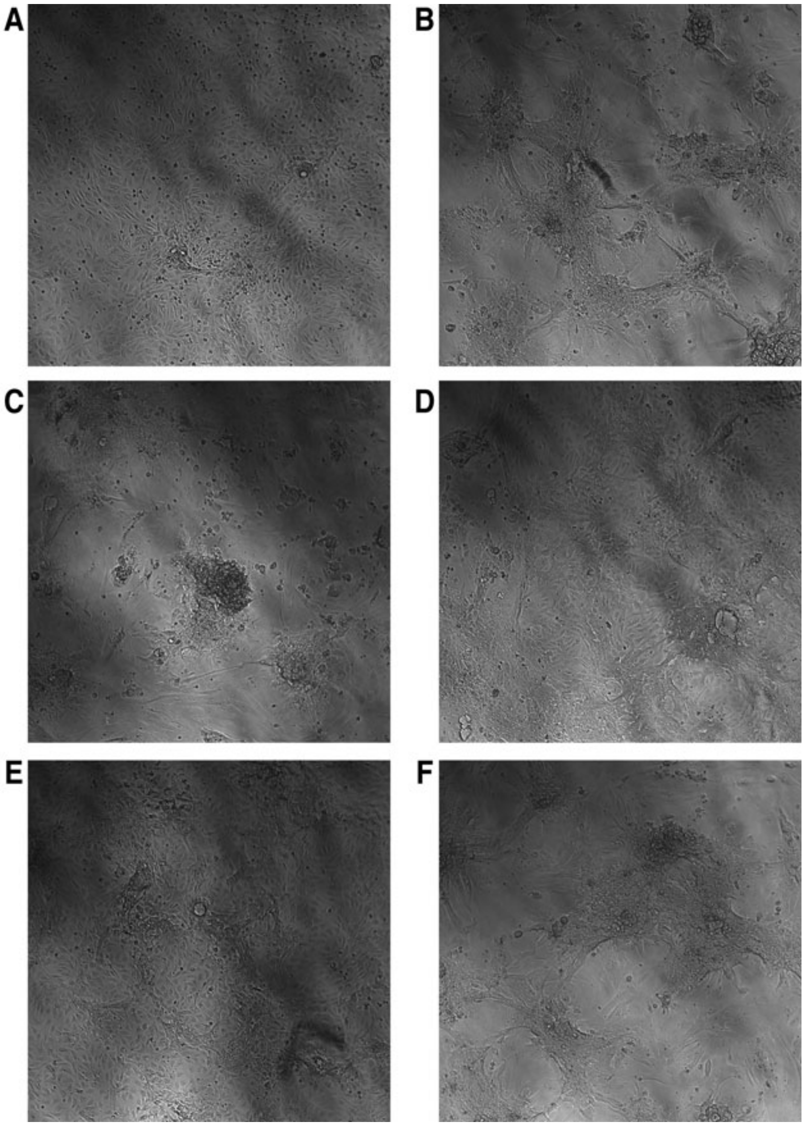

SUPPLEMENTARY FIG. S1. Sorted and replated non-CMs and human induced pluripotent stem cell-derived CMs maintain co-cultures. **(A–F)** Phase-contrast images of non-CMs **(A)**, co-culture with 1 part non-CMs to 9 parts CMs; 1:9) **(B)**, 3:7 co-culture **(C)**, 1:1 co-culture **(D)**, 7:3 co-culture **(E)**, and CMs **(F)** on day 40 of differentiation following day 10 sort. Scale bars represent 500 μm. CM, cardiomyocyte; non-CMs, non-cardiomyocytes.

database of reported disease associations, as described previously [S1,S2]. The majority of clones used had also been previously evaluated by the Etoposide Sensitivity Assay (ESA) to confirm hiPSC quality [S3].

Culture of dermal fibroblasts

Primary human dermal fibroblasts (dFBs; from stock of starting material for hiPSC reprogramming) were cultured in Dulbecco's modified Eagle's medium (DMEM) with GlutaMAX (ThermoFisher) plus 10% fetal bovine serum (FBS), 1% penicillin/streptomycin, and 1% sodium pyruvate. Media were changed every 2–3 days. TrypLE Express was used for dissociation of dFBs, which were subsequently combined with sorted human induced pluripotent stem cell-derived cardiomyocytes (hiPSC-CM) at ratios of 1:9 and 1:1 for coculture experiments.

Quantitative reverse transcription–polymerase chain reaction analysis

Cells were collected for quantitative reverse transcription–polymerase chain reaction (qRT-PCR) analysis immediately following day 10 single-cell isolation (unsorted), or immediately following day 10 sorting [non-cardiomyocyte (non-CM) and CM populations]. For confirmation of *GJA1* knockdown, cells were collected between days 24 and 40.

Total RNA was extracted using Trizol (Invitrogen) and column purified with a Qiagen RNeasy kit. RNA was reverse transcribed to complementary DNA (cDNA) using an iScript cDNA synthesis kit (Bio-Rad). qRT-PCR reactions were set up in a 384-well plate, using 15 ng of cDNA per reaction and primers purchased from IDT (Supplementary Table S1). PCR amplification was performed with TaqMan universal PCR master mix (Applied Biosystems) and a

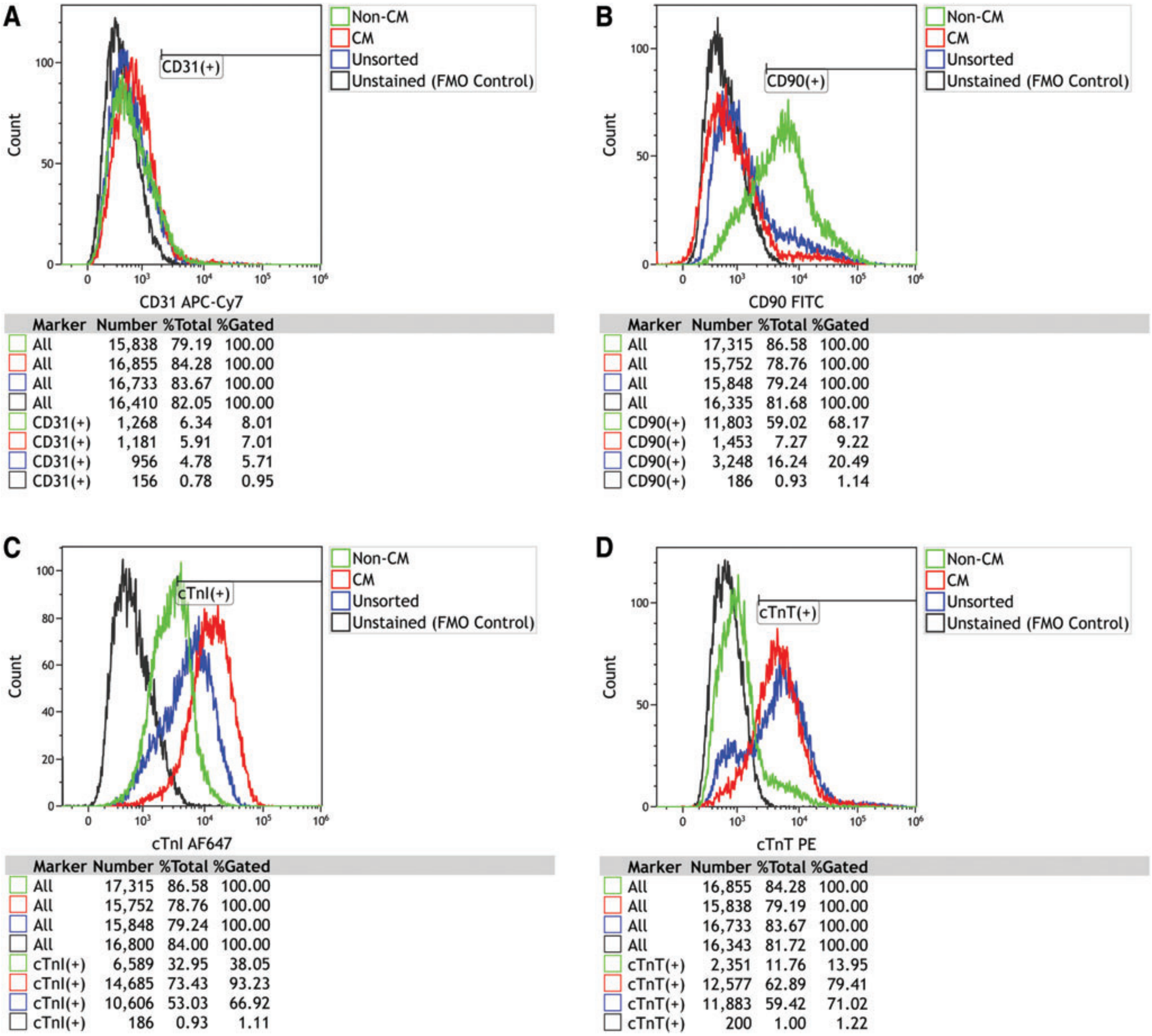

A

Marker	Number	%Total	%Gated
All	15,838	79.19	100.00
All	16,855	84.28	100.00
All	16,733	83.67	100.00
All	16,410	82.05	100.00
CD31(+)	1,268	6.34	8.01
CD31(+)	1,181	5.91	7.01
CD31(+)	956	4.78	5.71
CD31(+)	156	0.78	0.95

B

Marker	Number	%Total	%Gated
All	17,315	86.58	100.00
All	15,752	78.76	100.00
All	15,848	79.24	100.00
All	16,335	81.68	100.00
CD90(+)	11,803	59.02	68.17
CD90(+)	1,453	7.27	9.22
CD90(+)	3,248	16.24	20.49
CD90(+)	186	0.93	1.14

C

Marker	Number	%Total	%Gated
All	17,315	86.58	100.00
All	15,752	78.76	100.00
All	15,848	79.24	100.00
All	16,800	84.00	100.00
cTnI(+)	6,589	32.95	38.05
cTnI(+)	14,685	73.43	93.23
cTnI(+)	10,606	53.03	66.92
cTnI(+)	186	0.93	1.11

D

Marker	Number	%Total	%Gated
All	16,855	84.28	100.00
All	15,838	79.19	100.00
All	16,733	83.67	100.00
All	16,343	81.72	100.00
cTnT(+)	2,351	11.76	13.95
cTnT(+)	12,577	62.89	79.41
cTnT(+)	11,883	59.42	71.02
cTnT(+)	200	1.00	1.22

SUPPLEMENTARY FIG. S2. Analysis of cell-type markers in presorted and postsorted samples by flow cytometry. **(A–D)** Representative histograms for CD31 **(A)**, CD90 **(B)**, cTnI **(C)**, and cTnT **(D)** in unsorted, non-CM, and CM samples from the same differentiation. Events were gated on LIVE/DEAD staining and only live cells are plotted.

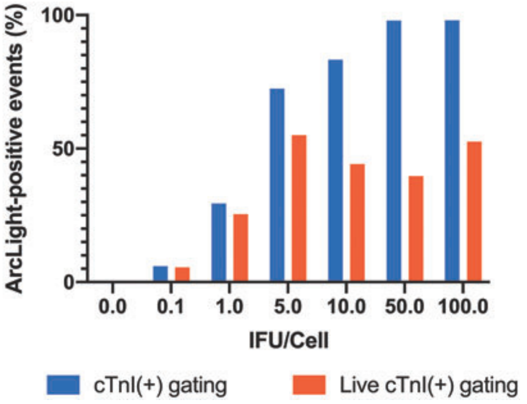

SUPPLEMENTARY FIG. S3. ArcLight lentiviral transduction efficiency for human induced pluripotent stem cell-derived CMs. Percentage of total cTnI(+) events (*blue*) or live cTnI(+) events (*red*), which were positive for ArcLight fluorescence versus lentiviral infectious units (IFU) per cell used for transduction. Day 20 spinner culture-derived CMs were plated at 100,000 cells per well of a 96-well plate, transduced with ArcLight lentivirus 4 days post-thaw, and analyzed by flow cytometry 72 h later for ArcLight expression, cTnI, and LIVE/DEAD stain. A total of five to eight wells were pooled per condition. IFU values per cell are assuming 50,000 adherent cells per well. 0 IFU/cell represents polybrene treatment only.

ViiA7 thermocycler (Applied Biosystems). All biological data points were generated from technical triplicates and analyzed by the ΔΔCt method, using *GAPDH* as the reference gene.

Flow cytometry

hiPSC-CMs and non-CMs were dissociated using the same approach as was applied before co-culture formation. Cells were washed in Dulbecco's phosphate-buffered saline (DPBS) and isolated using TrypLE Express. Viability staining was performed using LIVE/DEAD Fixable Violet Dead Cell Stain Kit (L34955; ThermoFisher), according to the manufacturer's instructions.

For extracellular staining, the cells were incubated for 30 min at 4°C in the dark with FACS buffer (0.5% FBS, 2 mM EDTA, 1×DPBS pH 7.4) containing FITC-conjugated CD90 antibody (clone 5E10; BD Biosciences), APC/Cy7-conjugated CD31 antibody (clone M89D3; BD Biosciences), isotype controls, or no antibody. The cells were washed with FACS buffer and fixed with 4% paraformaldehyde for 15 min at room temperature in the dark. Fixed cells were washed with FACS buffer, after which the protocol was either continued immediately or the cells were stored at 4°C in the dark. For experiments involving CD31 staining, fixation was performed with Stabilizing Fixative (338036; BD Biosciences).

Upon continuation of the protocol, the cells were permeabilized with BD Perm/Wash Buffer (from the Fixation/Permeabilization Solution Kit, 554714; BD Biosciences). For intracellular staining, the cells were incubated for 30 min at 4°C in the dark with Perm/Wash Buffer containing Alexa Fluor 647-conjugated cTnI antibody (clone C5; BD Biosciences), PE-conjugated cTnT antibody (clone 13–11; BD Biosciences), isotype controls, or no antibody. Fluorescence Minus One (FMO) controls were included for multiantibody panels. Cells were washed with Perm/Wash Buffer and assayed with a Beckman Coulter Gallios flow cytometer. Data analysis was performed using Kaluza flow cytometry analysis software (Beckman Coulter).

Immunofluorescence and microscopy

Cells were plated on fibronectin-coated (F1141; Sigma) Nunc Lab-Tek 8-well chamber slides (ThermoFisher) and fixed in 4% paraformaldehyde for 15 min. After fixation, they were washed three times with DPBS and stored at 4°C in DPBS until staining. The cells were subsequently permeabilized in 1% Triton X-100 for 30 min, washed three times with DPBS, and blocked for 3 h at room temperature with Super Block (ThermoFisher). The slides were then incubated with primary antibodies against cTnT (S-295-P0; ThermoFisher) and/or connexin 43 (Cx43; ab11369; Abcam) at dilutions of 1:200 or 1:500, respectively. Antibodies were diluted in DPBS containing 10% Super Block and 0.1% Tween. The slides were incubated overnight at 4°C on a rocker, and subsequently washed three times in a post-antibody wash buffer of DPBS plus 0.1% Tween.

They were then incubated for 1 h in the dark with Alexa Fluor 568-conjugated anti-mouse IgM, Alexa Fluor 488-conjugated anti-mouse IgG1, Alexa Fluor 488-conjugated anti-mouse IgM, or Alexa Fluor 633-conjugated anti-mouse IgG1 secondary antibodies (Invitrogen) diluted 1:500 in antibody dilution buffer. The slides were subsequently washed two times with post-antibody wash buffer, followed by two DPBS washes. Finally, they were incubated with DAPI in DPBS (Invitrogen) for 8 min, washed three times with DPBS, and washed once with distilled water. Slides were treated with 1–2 drops of Prolong Gold antifade (Invitrogen) and covered with 25 mm coverglass.

Stained slides were stored in the dark at 4°C until analysis using a Zeiss LSM 780 confocal microscope at 20×, 40×, or 100×. Images for analysis of nuclei count and cell size were acquired on a Nikon Eclipse Ti microscope using NIS-Elements imaging software. Cell area was determined from cTnT staining using "Freehand selection" and "Measure" tools in Fiji [S4,S5]. Live ArcLight- and RFP-expressing cells in co-cultures were imaged using a Zeiss LSM 780 confocal microscope at 10×. Phase-contrast images of sorted and replated CMs and non-CMs were obtained using a Zeiss Axiovert 40 CFL microscope at 10× and Zeiss Axiocam 503 mono camera.

Cloning and plasmid production

The *A242-ArcLight* plasmid (plasmid 36857; Addgene), Open Biosystems LentiORF pLEX-MCS Vector (Thermo Scientific), and SMARTvector lentiviral human *GJA1* or nontargeting control 1 CMV-TurboRFP short hairpin RNA (shRNA) plasmids (GE Healthcare) were grown in *Escherichia coli* cells and isolated with a plasmid maxi kit (Qiagen). The open reading frame for ArcLight was PCR amplified using KAPA HiFi HotStart PCR kit (Fisher Scientific) from the ArcLight plasmid, using primers (Supplementary Table S2) to add Not1 (forward) and Xho1 (reverse) restriction sites on either side. PCR products were run through an agarose gel and subsequently isolated using the QIAquick Gel Extraction Kit (Qiagen). The resulting PCR product was cloned into a TOPO TA Cloning Vector.

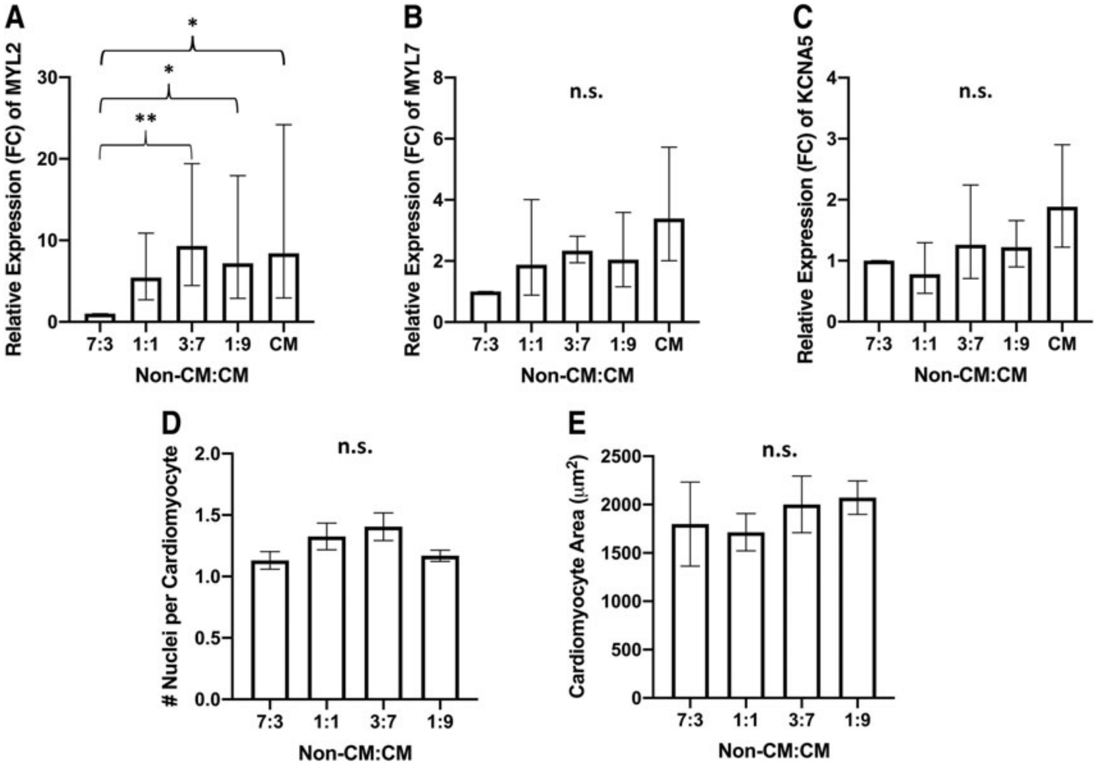

SUPPLEMENTARY FIG. S4. Gene expression and structural features of human induced pluripotent stem cell-derived CMs co-cultured with varying proportions of non-CMs. **(A–C)** Relative expression (FC) of *MYL2* **(A)**, *MYL7* **(B)**, and *KCNA5* **(C)** for CMs sorted out of co-cultures with 7 parts non-CMs to 3 parts CMs; 7:3), 1:1 co-cultures, 3:7 co-cultures, and 1:9 co-cultures, as analyzed by qRT-PCR. Samples were initially sorted for co-culture formation on day 10 of differentiation and resorted on day 40 to purify the CM fraction for analysis. CM denotes samples that were sorted on day 10 but not co-cultured. Error bars represent range of FC, calculated from standard deviation of ΔΔCt. Data are from four sorts representing two clones each from two unrelated individuals. *P* values were calculated from ΔΔCt values by ANOVA ($P < 0.01$ for MYL2 and n.s. for *MYL7* and *KCNA5*) followed by Bonferroni's test (*$P \leq 0.05$ adjusted value; **$P \leq 0.01$ adjusted value). **(D, E)**, Number of nuclei per CM **(D)** or CM cell area **(E)** for each co-culture condition, as determined by DAPI and cTnT staining of fixed cells on chamber slides. Three clones from unrelated individuals were analyzed between day 39 and 41 of differentiation. $n = 23–95$ cells **(D)** or 12–62 cells **(E)**. Error bars represent mean ± SEM. *P* values were calculated by Kruskal-Wallis test. ANOVA, analysis of variance; FC, fold change; n.s., not significant; qRT-PCR, quantitative reverse transcription–polymerase chain reaction; SEM, standard error of the mean.

The insert and full ArcLight insert sequence were verified by Sanger Sequencing (Supplementary Table S2) at the Mayo Clinic Medical Genome Facility. The ArcLight open reading frame was then excised using Not1 and Xho1 (New England BioLabs). The excised product was ligated into the LentiORF pLEX-MCS Vector and transformed into DH5-alpha competent bacteria. The final plasmid was isolated by plasmid maxi kit. The presence of the ArcLight insert in the LentiORF pLEX-MCS Vector was confirmed by restriction digest and gel electrophoresis, and further verified by Sanger Sequencing (Supplementary Table S2).

Lentiviral production and transduction

Lentiviral vectors were packaged in HEK293 cells using the Trans-Lentiviral Packaging Kit (Dharmicon) according to manufacturer's instructions, and then collected at 48 and 96 h after the low-serum medium change. Following centrifugation to separate the viral particle-containing supernatant from nonadherent cells and debris, viral particles were concentrated ∼50-fold with PEG-it Virus Precipitation Solution (System Biosciences), according to the manufacturer's instructions. Lentiviral titers were determined using a qRT-PCR lentiviral titer assay (MellGen Laboratories) and an Illumina Eco qPCR machine.

hiPSC-CMs were transduced using 150,000 infectious units (IFU) of ArcLight lentivirus per well of a 96-well plate in the presence of 8 μg/mL polybrene (Millipore Sigma), which was expected to fall within the optimal range for achieving maximal transduction efficiency, while maintaining CM viability (between 1 and 5 IFU per cell, Supplementary Fig. S3). Thawed and plated spinner culture-derived hiPSC-CMs were transduced at 8,000 IFU per well to produce similarly robust ArcLight expression without notable loss of viability. The cells were transduced between days 7 and 9 of differentiation (before D10 sort), or on day 22 (2 days post-thaw) for the spinner culture CMs used in the *GJA1* knockdown experiment (Fig. 6C–E).

For the SMARTvector shRNA lentiviruses, cells were transduced on day 7 of differentiation with a pool of three distinct shRNAs (100,000 IFU pooled shRNA/well) targeting *GJA1*. Cells transduced with shRNAs were visually verified to express an RFP reporter.

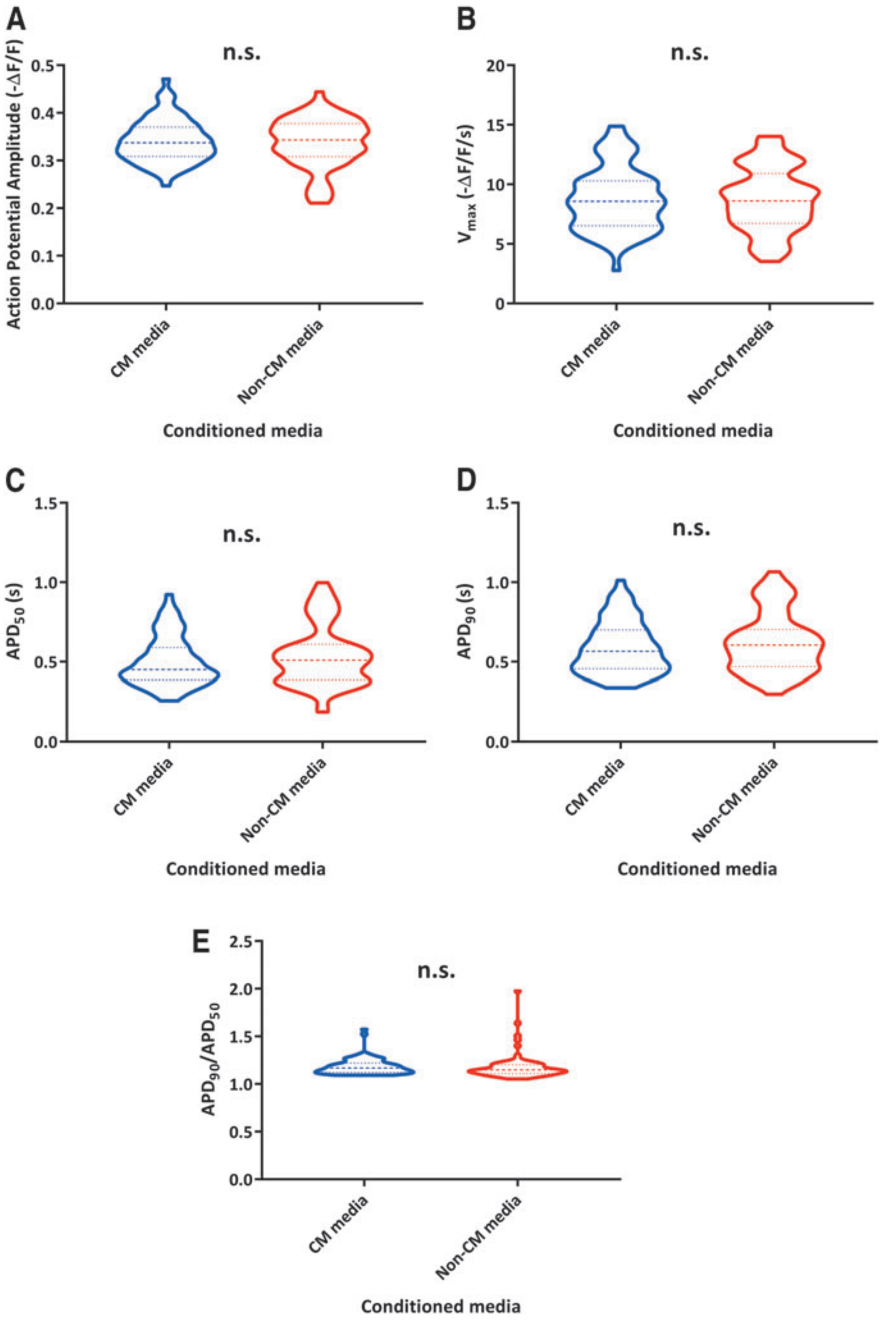

SUPPLEMENTARY FIG. S5. Non-CM- and CM-conditioned media do not differentially impact human induced pluripotent stem cell-derived CM electrophysiology after 2 weeks of co-culture. **(A–E)** Analysis of optical AP amplitude **(A)**, maximum upstroke velocities (V_{max}) **(B)**, APD_{50} **(C)**, APD_{90} **(D)**, and APD_{90}/APD_{50} **(E)** for CMs cultured with conditioned media from CMs or non-CMs. Data were collected from six independent differentiations representing five clones from two unrelated individuals between days 22 and 27 of differentiation. CM media: $n=70$ cells; non-CM media: $n=68$ cells. *Heavier dashed lines* within each violin indicate medians and *lighter dashed lines* indicate interquartile range. *P* values were calculated by either a Student's *t*-test (AP amplitude, V_{max}, APD_{90}) or Mann-Whitney U test (APD_{50}, APD_{90}/APD_{50}). AP, action potential; APD_{50}, action potential duration at 50% repolarization; APD_{90}, action potential duration at 90% repolarization.

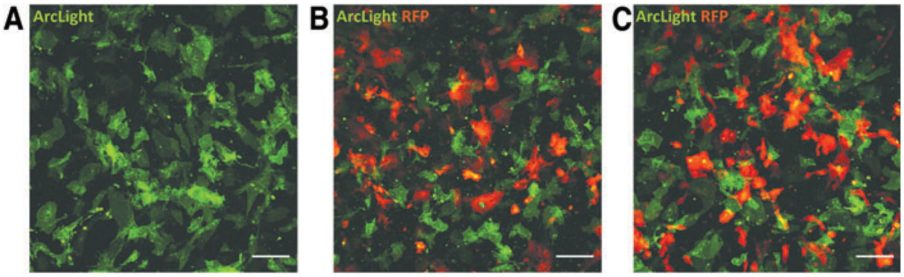

SUPPLEMENTARY FIG. S6. ArcLight-expressing human induced pluripotent stem cell-derived CMs and shRNA-expressing non-CMs plated together form co-cultures. **(A)** Representative fluorescent images of ArcLight-expressing (*green*) CMs alone. **(B, C)** ArLight-expressing CMs plated together at a 1:1 ratio with non-CMs transduced with either control **(B)** or *GJA1*-targeted **(C)** shRNAs (*red*) and imaged on day 39 of differentiation for the CMs, following 2 weeks of co-culture. Scale bar represents 200 μM. shRNA, short hairpin RNA.

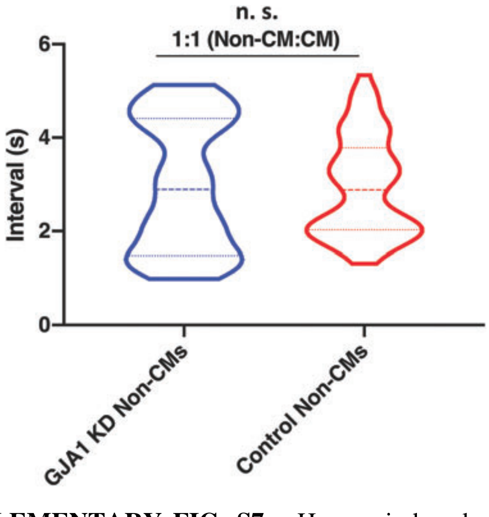

SUPPLEMENTARY FIG. S7. Human induced pluripotent stem cell-derived CMs co-cultured with *GJA1* knockdown or control non-CMs have similar beating rates. Interval between APs for spinner culture-derived CMs plated together in co-cultures at a 1:1 ratio with non-CMs transduced with either control or *GJA1*-targeted shRNAs. Data were collected in three independent experiments between days 38 and 40 for the CMs, following 2 weeks of co-culture. Analysis involved two clones in total (from distinct individuals) for both non-CMs and CMs. $n = 60$ cells per group. *Heavier dashed lines* within each violin indicate medians and *lighter dashed lines* indicate interquartile range. Data were analyzed by Mann-Whitney U test.

SUPPLEMENTARY TABLE S1. PRIMERS USED FOR QUANTITATIVE REVERSE TRANSCRIPTION–POLYMERASE CHAIN REACTION

Primer name	*Sequence ID*	*Exon region*
GAPDH	Hs.PT.39a.22214836	2–3
COL3A1	Hs.PT.58.4249241	1–2
POSTN	Hs.PT.58.4452022	4–5
THY1	Hs.PT.58.2281623	1–3
PDGFRB	Hs.PT.58.22892761	10–12
HCN4	Hs.PT.58.20502157	2–3
TNNT2	Hs.PT.58.25986741	2b–7a
TTN	Hs.PT.58.26848078.g	49–49
PLN	Hs.PT.58.23189767	1–2
IRX4	Hs.PT.58.20512314	1–2
HEY2	Hs.PT.58.582440	4–5
MYL2	Hs.PT.58.2578349	3–4
MYH7	Hs.PT.58.14589334	4–6
HEY1	Hs.PT.58.4299267	4–5
SLN	Hs.PT.58.23263368	1–2
NR2F2	Hs.PT.58.15681100	5–6
MYL7	Hs.PT.58.40405931	5–7
MYH6	Hs.PT.58.2106207	14–15
SCN5A	Hs.PT.58.28073583	16–17
CACNA1C	Hs.PT.58.14979004	30–31
KCNJ2	Hs.PT.58.27651196	1–2
KCNA5	Hs.PT.58.2224557	1a–1a
GJA1	Hs.PT.58.38338544	1–2

Supplementary Table S2. Primers Used for ArcLight Cloning

Purpose	*Descriptor*	*Sequence*
Add restriction sites to ArcLight open reading frame	Forward	5′-ATTATAGCGGCCGCATGGAGGGATTCGACGG-3′
	Reverse	5′-GCGCCCCTCGAGTCATTTGTATAGTTCATCCATGC-3′
Confirm ArcLight open reading frame sequence and insertion into TOPO TA Cloning Vector	M13 forward	5′-GTAAAACGACGGCCAG-3′
	M13 reverse	5′-CAGGAAACAGCTATGAC-3′
	ArcLight internal forward	5′-CGGGATGGCTTTGGCTCTTTC-3′
	ArcLight internal forward	5′-TTTCAAGAGTGCCATGCCCG-3′
	ArcLight internal reverse	5′-CCTTCGGGCATGGCACTCTTG-3′
	ArcLight internal reverse	5′-CCCGTAGGCAAATATCCTTAATCC-3′
Confirm ArcLight open reading frame insertion into LentiORF pLEX-MCS Vector	Forward	5′-CACCAAAATCAACGGGACTT-3′
	Reverse	5′-ATATAGACAAACGCACACCGGCCT-3′

Supplementary References

S1. Perales-Clemente E, AN Cook, JM Evans, S Roellinger, F Secreto, V Emmanuele, D Oglesbee, VK Mootha, M Hirano, et al. (2016). Natural underlying mtDNA heteroplasmy as a potential source of intra-person hiPSC variability. EMBO J 35:1979–1990.

S2. Lott MT, JN Leipzig, O Derbeneva, HM Xie, D Chalkia, M Sarmady, V Procaccio and DC Wallace. (2013). mtDNA variation and analysis using mitomap and mitomaster. Curr Protoc Bioinformatics 44:1 23 1–26.

S3. Secreto FJ, X Li, AJ Smith, ES Bruinsma, E Perales-Clemente, S Oommen, G Hawse, SCL Hrstka, BK Arendt, et al. (2017). Quantification of etoposide hypersensitivity: a sensitive, functional method for assessing pluripotent stem cell quality. Stem Cells Transl Med 6:1829–1839.

S4. Schneider CA, WS Rasband and KW Eliceiri. (2012). NIH image to ImageJ: 25 years of image analysis. Nat Methods 9:671–675.

S5. Schindelin J, I Arganda-Carreras and E Frise. (2012). Fiji: an open source platform for biological-image analysis. Nat Methods 9:676–682.
